# A Systematic Screening of ADHD-Susceptible Variants From 25 Chinese Parents–Offspring Trios

**DOI:** 10.3389/fgene.2022.878036

**Published:** 2022-04-26

**Authors:** Qianqian Li, Yingying Meng, Jingyang Wang, Yuhang Xie, Tian Li, Wei Sun

**Affiliations:** ^1^ Department of Psychological Counseling, Second Affiliated Hospital of Chongqing Medical University, Chongqing, China; ^2^ School of Medicine, Nankai University, Tianjin, China; ^3^ State Key Laboratory of Silkworm Genome Biology, Southwest University, Chongqing, China

**Keywords:** ADHD, WGS, SNV, complex disorders, susceptible

## Abstract

Attention-deficit/hyperactivity disorder (ADHD) is one of the most prevalent and heritable childhood behavioral disorders. Although a number of ADHD-susceptible regions had been identified, details about the variations of genes and their related patterns involved in ADHD are still lacking. In this study, we collected 25 Chinese parents–offspring trios, each of which consisted of a child diagnosed with ADHD and his/her unaffected parents, and analyzed the variations from whole-genome sequencing data. SNVs in reported ADHD-susceptible regions and on the genes whose functions were related to dopamine were screened, and we identified a set of variants with functional annotations which were specifically detected in ADHD children, including most SNVs in the gene coding region that might impair protein functions and a few SNVs in promoter or 3′ untranslated region (3′-UTR) that might affect the regulation of relative gene expression in a transcriptional or posttranscriptional level. All the information may further contribute to the understanding, prediction, prevention, and treatment of ADHD in clinical.

## Introduction

Attention-deficit/hyperactivity disorder (ADHD) is one of the most prevalent and heritable childhood behavioral disorders, which affects 2%–6% of school-age children (Bakker et al., 2003; Ford et al., 2003). ADHD is typically characterized by inattention, excessive motor activity, impulsivity, and distractibility (Hawi et al., 2005). People with ADHD are at risk for a wide range of functional impairments: school failure, peer rejection, injuries due to accidents, criminal behavior, occupational failure, divorce, suicide, and premature death (Faraone and Larsson, 2019). It has been estimated that at least 15% of children diagnosed with ADHD (childhood ADHD) will continue to retain a full diagnosis by the age of 25, approximately 40% will show just partial remission and continue to experience impairing symptoms, and only 40% will get a complete remission (Franke et al., 2018). ADHD is commonly assumed to be heterogeneous and multifactorial in genetics, with the involvement of many environmental risk factors, including prenatal and perinatal events, environmental toxins, and dietary and psychosocial stimuli (Thapar et al., 2013). However, the underlying etiological mechanisms of ADHD remain largely unclear.

Classical genetic studies indicate that ADHD is strongly heritable, with an estimated heritability for childhood ADHD on an average of 75% (Faraone et al., 2005). In the first genome-wide scan for ADHD, four chromosomal regions, 5p12, 10q26, 12q23, and 16p13, were suggested to be possibly susceptible regions by the multipoint maximum LOD scores (MLSs) greater than 1.5, but no region exceeded the criterion for significant or suggestive linkage (Fisher et al., 2002). Another independent group (Ogdie et al., 2004) accomplished a genome-wide scan of 308 affected sibling pairs and presented strong evidence for four susceptible chromosomal locations (5p13, 6q12, 16p13, and 17p11), with an overlap of only one nominally significant region (16p13) with the previous report. Moreover, Ogdie et al. (2003) and Smalley et al. (2002) performed linkage analyses on a same 270 affected sib-pair cohort and identified suggestive ADHD linkage for 17p11 (MLS = 2.98) and four other regions with MLS values greater than 1.0, including 5p13, 6q14, 11q25, and 20q13. Moreover, a most recent GWAS-based meta-analysis on 20,183 individuals diagnosed with ADHD and 35,191 controls identified additionally 12 independent ADHD risk loci in the genetic background of the European population (Demontis et al., 2019).

Apparently, the genetic basis of ADHD is highly heterogeneous. Although a number of ADHD-susceptible regions had been identified with more or less overlaps in several studies, details of the variations of genes and their related patterns involved in ADHD, particular in East Asian populations, are still lacking. In this study, a cohort of 25 Chinese parents–offspring trios, in which each trio consisted of a child diagnosed with ADHD and his/her unaffected parents, were collected, and whole-genome sequencing (WGS) was performed; a set of novel variants associated with ADHD were identified through the systematic screening on this cohort.

## Materials and Methods

### Sample Collection

The cohort consisted of 25 parents–offspring trios. Within each trio, the child was clinically diagnosed as an ADHD patient, while his/her parents were presented with no history of ADHD. All samples were collected from West China Mental Health Center. This study was approved by the Ethics Committee of Chongqing Ninth People’s Hospital. Informed consent for DNA analysis was obtained from each family in line with local institutional review board requirements at the time of collection.

### DNA Extraction, Library Construction, and Sequencing

Genomic DNA extracted from peripheral blood of each sample was fragmented to an average size of ∼350 bp and subjected to DNA library construction using established Illumina paired-end protocols. The Illumina Novaseq 6000 platform (Illumina Inc., San Diego, CA, United States) was utilized for genomic DNA sequencing in Novogene Bioinformatics Technology Co., Ltd. (Beijing, China) to generate 150-bp paired-end reads, with a minimum coverage of 10× for ∼90% of the genome (mean coverage of 30×).

### Raw Sequencing Data Processing

After sequencing, conversion and demultiplexing of Illumina Base Call Files (bcl files) were performed with bcl2fastq software (Illumina). The resulting fastq data were submitted to in-house quality control software for removing low-quality reads and then were aligned to the reference human genome (GRCh37/hg19) using the Burrows–Wheeler Aligner (Li and Durbin, 2009), and duplicate reads were marked using Sambamba (Tarasov et al., 2015).

### SNV/Indel Calling and Annotation

Single-nucleotide variants (SNVs) and indels were called with Samtools (Li et al., 2009) to generate gVCF files. Annotation was performed using ANNOVAR (8 June 2017) (Wang et al., 2010). The annotations included minor allele frequencies from public databases, as well as deleteriousness and conservation scores enabling further filtering and assessment of the variants. The influence of variants on protein functions was predicted by the Polyphen2_HDIV algorithm (for multifactorial disorders) based on the HumanDiv database. The influence of variants on miRNA regulatory sites was annotated with the TargetScanS database, and the influence of variants on transcriptional factor (TF) regulatory sites was annotated with the TRANSFAC database.

### Other Public Data Source and Data Plotting

Human brain mRNA and miRNA expression data were downloaded from ENCODE (https://www.encodeproject.org/). Gene enrichment assays were performed with the R clusterProfiler package (Yu et al., 2012). Plots were drawn by ggplot2.

## Results

### Features of Variants in Reported ADHD-Susceptible Regions

**FIGURE 1:**
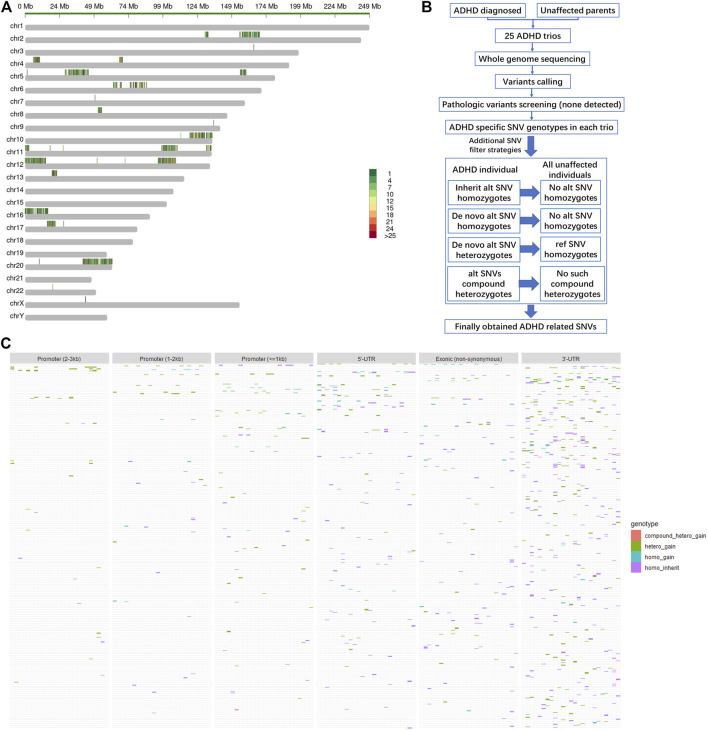
Distribution of filtered SNVs in the reported ADHD-susceptible regions. **(A)** A map showing the reported ADHD-susceptible regions in the human genome and the distribution of the variations in these regions in the cohort. Color annotation: red to green, high to low frequency detected in the cohort. **(B)** A diagram depicting the strategies for SNV filtering in the cohort. **(C)** Heatmaps showing the distribution of filtered SNVs in ADHD children as well as in genomic regions including promoters, 5′-UTR, coding region (exon), and 3′-UTR. Genotype categories of each site were labeled in different colors as indicated in the legend.

As expected, none of the 25 ADHD children in the cohort carried any explicit pathologic or likely pathologic variants. Alternatively, the frequencies of all single-nucleotide variants (SNVs) located in the reported ADHD-susceptible regions in the human genome are shown in Figure 1A, in which red and green indicate high and low frequencies, respectively. Moreover, genes in these regions with the expression level lower than RPKM = 1 in the human brain (based on ENCODE dataset) were considered very low expressed, and SNVs on these genes were dropped for their limited functional contribution in the central nerve system (Supplementary Figure S1A). Then the retained SNVs were further filtered based on the rules shown in Figure 1B. Only those SNVs that were *de novo* gained (homo_gain, hetero_gain or compound hetero_gain) or form homozygotes (homo_inherit) in ADHD children, but were not found in certain genotypes of unaffected parents in the whole cohort, were kept and considered as the potentially ADHD-susceptible sites. The distribution of these sites in gene body regions and regulatory regions (promoters) is shown in Figure 1C.

**FIGURE 2:**
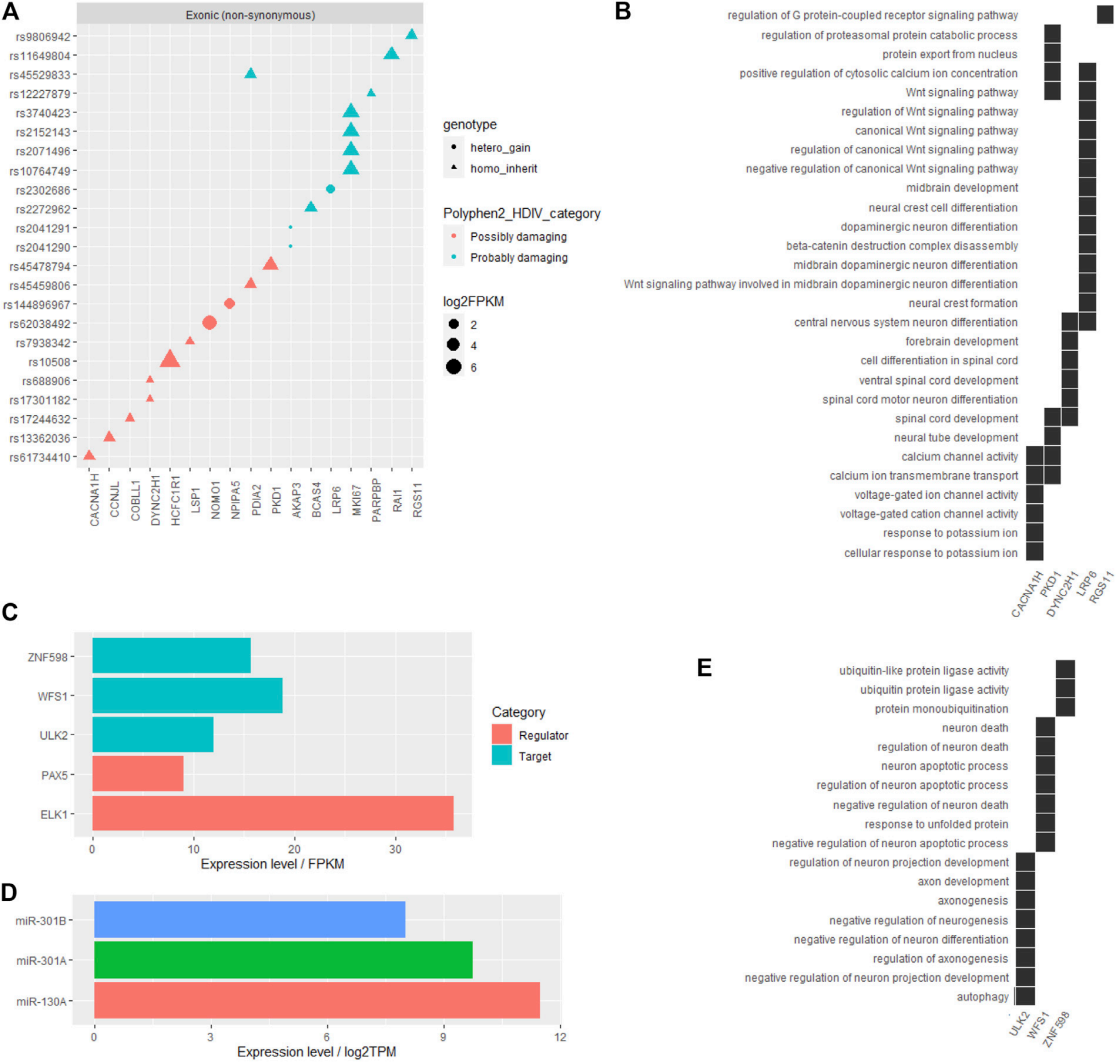
Functional annotation on the filtered SNVs in the reported ADHD-susceptible regions. **(A)** A dot plot showing the information of filtered SNVs in coding regions and their corresponding genes. SNV genotype, influence on protein functions, and gene expression level were marked by dot shape, color, and size, respectively. **(B)** A heat plot showing the distribution of certain genes on neuron-related functions potentially involved in ADHD. **(C)** Bar plot showing the expression levels of certain genes, including the genes corresponding to functional annotated SNVs in the promoter or 3′-UTR and their relative regulators in human brain tissue. **(D)** Bar plot showing the expression levels of miRNAs that targeted the site affected by the identified SNV. **(E)** A heat plot showing the distribution of certain genes corresponding to SNVs in the promoter or 3′-UTR on neuron-related functions.

Among all the filtered SNVs, the influence of gene coding region (exons) variations on protein functions was predicted by the Polyphen2_DHIV program and annotated as benign, possibly damaging, or probably damaging (Supplementary Figure S1B). Genes corresponding to those SNVs labeled as possibly damaging or probably damaging are shown in Figure 2A, and part of these genes, including CACNA1H, PKD1, DYNC2H1, LRP6, and RGS11, played primarily neuron-related functions that might contribute to ADHD, such as dopaminergic neuron differentiation, midbrain development, ion channel activity, and Wnt signaling pathway (Figure 2B). Other detailed information of these obtained coding region SNVs are listed in Table 1.

**TABLE 1:** Information of filtered SNVs in the coding region of genes in reported ADHD-susceptible regions.

SNV_ID	Gene	CytoBand	Genotype	Ref	Alt	AAChange	X1000g_Chinese	X1000g_EAST	Polyphen2_HDIV_category	Frequency_in_cohort	Sample
rs17244632	COBLL1	2q24.3	homo_inherit	G	A	p.T833I	0.044851	0.0327	Possibly damaging	1/25	75
rs13362036	CCNJL	5q33.3	homo_inherit	G	A	p.H186Y	0.164452	0.1627	Possibly damaging	2/25	6/100
rs10764749	MKI67	10q26.2	homo_inherit	C	T	p.R2426Q	0.104651	0.1181	Probably damaging	1/25	100
rs2071496	MKI67	10q26.2	homo_inherit	G	C	p.T2508S	0.104651	0.119	Probably damaging	1/25	100
rs2152143	MKI67	10q26.2	homo_inherit	C	T	p.G682S	0.104651	0.1181	Probably damaging	1/25	100
rs3740423	MKI67	10q26.2	homo_inherit	T	A	p.E1043V	0.104651	0.1181	Probably damaging	1/25	100
rs7938342	LSP1	11p15.5	homo_inherit	T	A	p.H34Q	0.767442	0.7688	Possibly damaging	1/25	86
rs17301,182	DYNC2H1	11q22.3	homo_inherit	C	T	p.H341Y	0.086379	0.0933	Possibly damaging	1/25	5
rs688906	DYNC2H1	11q22.3	homo_inherit	A	G	p.K1413R	0.782392	0.7827	Possibly damaging	2/25	71/79
rs2302686	LRP6	12p13.2	hetero_gain	G	C	p.S817C	0.009967	0.0109	Probably damaging	1/25	5
rs2041290	AKAP3	12p13.32	hetero_gain	A	G	p.S700L	0.126246	0.1647	Probably damaging	1/25	65
rs2041291	AKAP3	12p13.32	hetero_gain	G	A		0.126246	0.1647	Probably damaging	1/25	65
rs12227879	PARPBP	12q23.2	homo_inherit	G	A	p.V319M	0.033223	0.0327	Probably damaging	1/25	42
rs144896967	NPIPA5	16p13.11	hetero_gain	C	T	p.E268K	0.023256	0.0268	Possibly damaging	1/25	12
rs62038492	NOMO1	16p13.11	hetero_gain	A	G	p.E1153G	NA	NA	Possibly damaging	1/25	74
rs10508	HCFC1R1	16p13.3	homo_inherit	G	T	p.P73Q	0.109635	0.1181	Possibly damaging	1/25	98
rs45459806	PDIA2	16p13.3	homo_inherit	C	T	p.A316V	0.018272	0.0198	Possibly damaging	1/25	100
rs45478794	PKD1	16p13.3	homo_inherit	G	A	p.T3509M	0.068106	0.0625	Possibly damaging	1/25	2
rs45529833	PDIA2	16p13.3	homo_inherit	C	G	p.P382A	0.021595	0.0218	Probably damaging	1/25	100
rs61734410	CACNA1H	16p13.3	homo_inherit	C	T	p.P640L	0.835548	0.8383	Possibly damaging	2/25	99/100
rs9806942	RGS11	16p13.3	homo_inherit	C	T	p.V167M	0.325581	0.3185	Probably damaging	1/25	100
rs11649804	RAI1	17p11.2	homo_inherit	C	A	p.P165T	0.845515	0.8353	Probably damaging	1/25	100
rs2272962	BCAS4	20q13.13	homo_inherit	G	T	p.E56D	0.131229	0.1329	Probably damaging	1/25	75

Besides coding regions, variants in 3′-UTR (3′ untranslated region) may interfere with the motifs for mRNA–miRNA mutual recognition and variants in promoter region may change the *cis-*element recognized by certain transcriptional regulators. Among all filtered SNVs in this cohort, only two variants in the promoter regions and one variant in 3′-UTR were annotated to impact certain *cis*-elements (Supplementary Figure S1C) and the miRNA-binding site (Supplementary Figure S1D), respectively. Notably, all these SNVs corresponding genes, including ZNF598, WFS1, and ULK2, as well as their regulator PAX5, ELK1, miR-130A, and miR-301A/B, showed a moderate to high expression level in the human brain (Figures 2C,D), suggesting the potential involvement of these regulatory patterns in ADHD pathogenesis. Moreover, the regulatory target genes ZNF598, WFS1, and ULK2 primarily played roles in protein ubiquitination, neuron death, and axonogenesis, all of which may contribute to ADHD (Figure 2E). Other detailed information of this part of SNVs is listed in Table 2.

**TABLE 2:** Information of filtered SNVs in the promoter region and 3′-UTR of genes in the reported ADHD-susceptible regions.

SNV_ID	Gene	Cytoband	Genotype	Ref	Alt	X1000g_Chinese	X1000g_EAST	Region	TF/miRNA_Sites	Frequency_in_cohort	Sample
rs67529412	ZNF598	16p13.3	homo_gain	C	G	NA	NA	Promoter	ELK1_01	1/25	8
rs866882393	WFS1	4p16.1	hetero_gain	G	C	NA	NA	Promoter	PAX5_01	3/25	42/65/78
rs117345841	ULK2	17p11.2	hetero_gain	T	C	0.064784	0.0665	3'-UTR	miR-130/301	1/25	65

### Features of Variants in Other Dopamine-Related Genes

**FIGURE 3:**
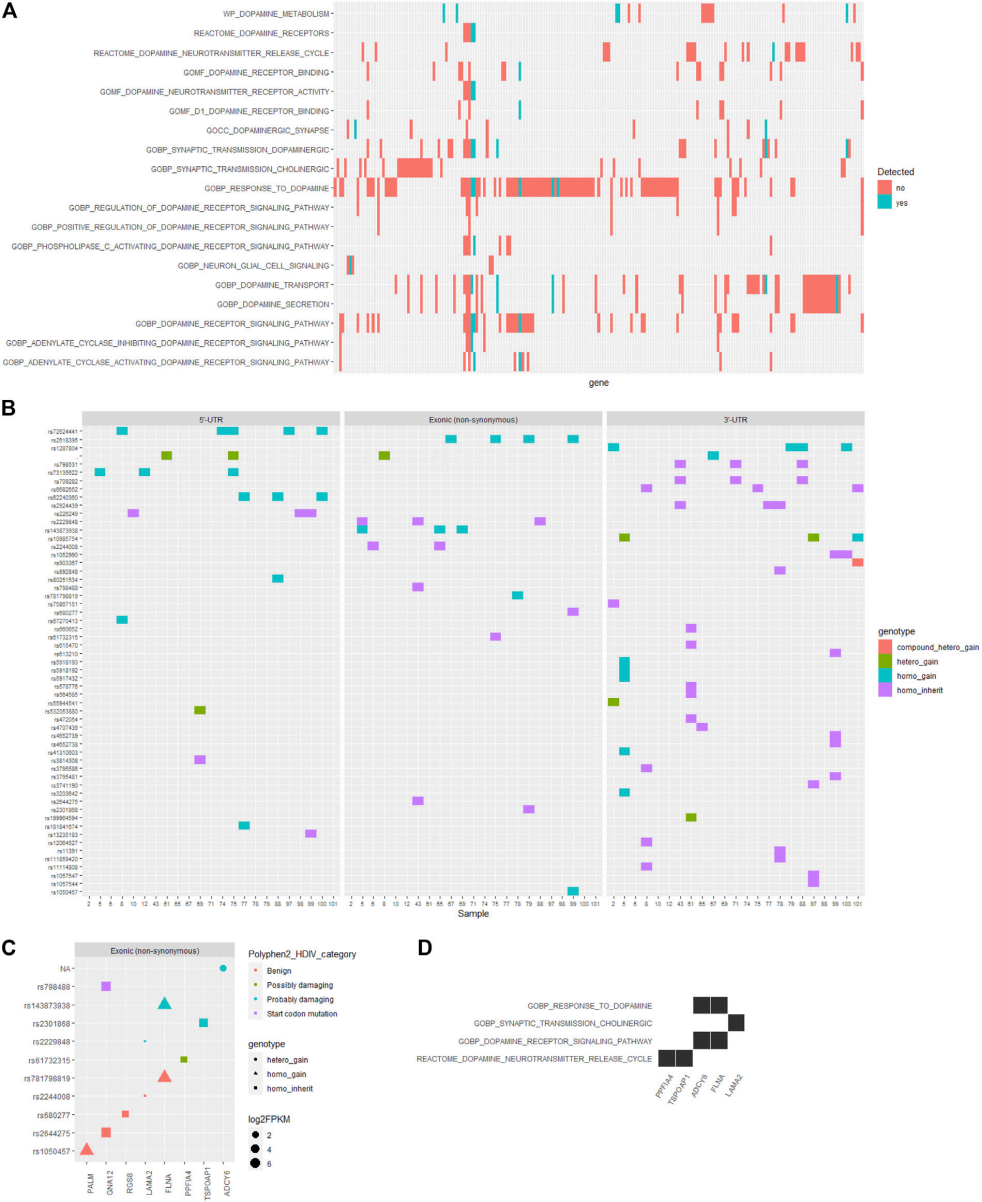
Distribution and functional annotation of filtered SNVs in dopamine-related genes. **(A)** A heatmap showing the distribution of intersect genes on dopamine-related functions and in the reported ADHD-susceptible regions (colored in cyan). **(B)** Heatmaps showing the distribution of filtered SNVs locating in dopamine-related genes in ADHD children and in genomic regions including 5′-UTR, coding region (exon), and 3′-UTR. Genotype categories of each site were labeled in different colors as indicated in the legend. **(C)** A dot plot showing the information of filtered SNVs and their corresponding genes. SNV genotype and the influence on protein functions, and gene expression level were marked by dot shape, color, and size, respectively. **(D)** A heat plot showing the distribution of certain genes corresponding to SNVs in coding regions on dopamine-related functions.

On the other hand, the pathogenesis of ADHD has been closely associated with the dopamine-related processes (Kahn et al., 2003; Zhou et al., 2010). Herein, we examined the functional annotations of genes corresponding to those filtered SNVs in the reported ADHD-susceptible regions and found that among the whole set of genes participating in dopamine-related processes as annotated by the Gene Ontology and Reactome database, only a small section located the reported ADHD-susceptible regions (Figure 3A, Supplementary Figure S2A). Following the identical criteria used for SNV filtering, we obtained an additional set of SNVs that were corresponding to the genes involved in dopamine-related processes while were not in reported ADHD-susceptible regions, and the distribution of these SNVs in gene body regions is shown in Figure 3B. Accordingly, the influence of these coding region (exons) variations on their corresponding genes was predicted by Polyphen2_DHIV, and notably, there was a variation (rs798488) in GNA12 causing the start codon mutation that led to none or N-terminal truncated protein translation (Figure 3C). In addition, genes corresponding to those SNVs predicted as possibly or probably damaging contained PPFIA4, TSPOAP1, ADCY6, FLNA, and LAMA2, and these genes were primarily involved in the process of dopamine neurotransmitter release and dopamine receptor signaling (Figure 3D). On the other hand, no SNVs in 3′-UTR (Supplementary Figure S2B) or promoter regions (Supplementary Figure S2C, D) were annotated to have an impact on any known *cis*-elements or miRNA-targeting sites. Detailed information of this part of SNVs is listed in Table 3.

**TABLE 3:** Information of filtered SNVs in the coding region of dopamine-related genes.

SNV_ID	Gene	Cytoband	Genotype	Ref	Alt	AAChange	X1000g_Chinese	X1000g_EAST	Polyphen2_HDIV_category	Frequency_in_cohort	Sample
rs680277	RGS8	1q25.3	homo_inherit	T	C	p.N3S	0.146179	0.1399	Benign	1/25	99
rs61732315	PPFIA4	1q32.1	homo_inherit	G	T	p.A825S	0.171096	0.1577	Possibly damaging	1/25	75
rs2229848	LAMA2	6q22.33	homo_inherit	C	T	p.A2583V	0.621262	0.6369	Probably damaging	3/25	5/43/88
rs2244008	LAMA2	6q22.33	homo_inherit	A	G	p.T2632A	0.124585	0.1319	Benign	2/25	6/65
rs2644275	GNA12	7p22.2	homo_inherit	G	A	p.T23I	0.222591	0.2321	Benign	1/25	43
rs798488	GNA12	7p22.2	homo_inherit	T	C	p.M1V	0.219269	0.2232	Start codon mutation	1/25	43
NA	ADCY6	12q13.12	hetero_gain	G	A	p.L329F	NA	NA	Probably damaging	1/25	8
rs2301868	TSPOAP1	17q22	homo_inherit	C	T	p.G1770E	0.144518	0.1161	Probably damaging	1/25	79
rs1050457	PALM	19p13.3	homo_gain	A	G	p.T107A	0.534884	0.5446	Benign	1/25	99
rs143873938	FLNA	Xq28	homo_gain	C	T	p.V528M	0.047826	0.0419	Probably damaging	3/25	5/65/69
rs781798819	FLNA	Xq28	homo_gain	T	C	p.K676R	0.006522	0.0065	Benign	1/25	78

## Discussion

Attention-deficit/hyperactivity disorder (ADHD) is generally described as a multifactorial genetic disorder; however, the genetic basis of its pathogenesis is less studied. Although a set of susceptible regions have been identified based on linkage analysis in several independent cohorts, such as 4p16.1, 5p12-13, 6q12-14, 10q26, 11p15.5, 11q25, 12q23, 16p13, 17p11, and 20q13 (Fisher et al., 2002; Smalley et al., 2002; Ogdie et al., 2003; Ogdie et al., 2004; Faraone et al., 2005), the knowledge of exact genes and variants involved in ADHD is still lacking. In this study, we collected 25 unrelated parents–offspring trios in which only the 25 children were diagnosed with ADHD, and called the variants from the whole-genome sequencing (WGS) data of all 75 samples. The SNV filtering strategy used for this study is based on the hypothesis that *de novo* and homozygous variants specifically detected in ADHD children are more likely to contribute to the pathogenesis of ADHD. With such criteria, we screened the SNVs in two sections: the SNVs in the reported ADHD-susceptible regions and the SNVs on genes that play dopamine-related functions (Kahn et al., 2003; Zhou et al., 2010). Among all obtained variants, SNVs in 3′-UTR account for the majority. However, few of them showed influence on gene expression based on mRNA–miRNA interaction. The situation of SNVs in promoter regions is similar. In contrast, a much higher number of SNVs in the gene coding region showed a potential impact on protein functions, although the total amount of these SNVs is less. Another feature of these filtered SNVs is that recurrent SNVs in 25 ADHD children in the cohort are common, but functional annotated SNVs are few and rarely recurrent, partially due to the relatively low frequency of *de novo* variation events.

In this study, identified functional SNVs in promoter regions contain rs67529412 and rs866882393 corresponding to ZNF598 and WFS1, respectively. Notably, both of these sites were *de novo* variations in this cohort, and allele frequency (AF) of neither site was included in the Chinese or East Asian population in the 1000 Genomes database, implying the very low frequency of these variants. Particularly, the variant of rs866882393, which eliminates the PAX5-binding motif in the promoter region of WFS1, was recurrently detected in three ADHD children, and it has been reported that the expression of WFS1 is closely correlated with neuronal differentiation (Tekko et al., 2014; Li et al., 2020). rs117345841 is the uniquely identified functional SNV in 3′-UTR and has a low AF of approximate 0.06 in the Chinese population in the 1000 Genomes database, and the variation in this site impairs the ULK2-miR-130A/miR-301A/B interaction. Notably, the function of ULK2 is related to neuron axon development.

Identified functional SNVs in gene coding regions that may impair protein functions are many. Among these sites, corresponding genes, CACNA1H, PKD1, DYNC2H1, LRP6, PPFIA4, TSPOAP1, ADCY6, FLNA, and LAMA2, are annotated to play central nervous system-related functions. Particularly, defects in synapse formation and function lead to various neurological diseases, and a recent study suggests that protein PKD1 functions upstream of N-cadherin, a classical synaptic adhesion molecule, to promote functional synapse formation (Cen et al., 2018). CACNA1H is also highly associated with mental disorders. A genome-wide analysis based on 232,964 cases and 494,162 control for eight mental disorders including ADHD suggested that polygenic risk sites of interest were enriched in genes previously associated with neuroticism, cognitive ability, and nocturnal sleep phenotypes, and CACNA1I is one of the genes associated with cognitive ability (Cross-Disorder Group of the Psychiatric Genomics Consortium, 2019). Han et al. (2018) found that 10 of 35 patients, all of whom were diagnosed differently with developmental delay (DD) and/or intellectual disability (ID), were found to have underlying genetic etiology and carried autosomal dominant inheritance of nine gene mutations, including a heterozygous missense mutation (c.5675G > A; p. R1892H) of the CACNA1H identified in a 13-year-old male patient with intermittent epileptic discharges in the right temporal areas and an overall IQ of 68 (mild ID). Moreover, MTF1-activated CACNA1H transcription in COCH (coagulation factor c homolog) neurons encodes the ability to burst action potentials and cause social stress–induced anxiety-like behaviors by synapsing directly with a subset of GABAergic inhibitory neurons in the lateral septum (Jing et al., 2021), and CACNA1H was also identified as a susceptibility gene in amyotrophic lateral sclerosis (ALS) (Rzhepetskyy et al., 2016). In addition, Wnt/LRP6 signaling is a key regulator of axonal remodeling, synaptic plasticity, neurite growth, and *β*-catenin-independent neurotransmitter release (Acebron and Niehrs, 2016), and a genome-wide linkage study has defined a broad susceptibility region of late-onset Alzheimer’s disease on chromosome 12, which contains the LRP6 gene (De Ferrari et al., 2007). Moreover, rs2302686 in LRP6 and a variant in ADCY6 are *de novo* variations, and the gene functions are related to the dopaminergic neuron development and the dopamine receptor signaling pathway, respectively.

Although this study cannot give the full view of the genetic basis of ADHD, it provides a series of novel insights for the understanding of ADHD in the Chinese population. Taken together, in this study, in a cohort containing 25 ADHD trios, we identified a set of SNV variations specifically in ADHD children and annotated to be functional for gene expression regulation or protein function, and linked some of these sites to the reported ADHD-susceptible regions or dopamine-related functions based on the annotation of their corresponding genes. All the information may further contribute to the understanding, prediction, prevention, and treatment of ADHD in clinical.

## Data Availability

The data presented in the study are deposited in the BioDB Platform repository, accession: https://biodb.org/download/genome/human/X101SC20114684-Z01-J003_B3S75_20210108.
