# Supplementary material for: A Systematic Screening of ADHD-Susceptible Variants From 25 Chinese Parents–Offspring Trios
Source: Front Genet. 2022 Apr 26;13:878036. doi: 10.3389/fgene.2022.878036 (PMC9087589; doi:10.3389/fgene.2022.878036)
Supplement: Supplementary file 1 [file Presentation1.pdf]

Supplementary figure legends

Fig.S1 Additional information of the distribution and annotation of filtered SNVs in reported ADHD susceptible region. (A) A bar plot showing the expression value of genes in reported ADHD susceptible regions. Genes on different chromosomes were marked in different colors, the dashed line indicated the criterion of FPKM=1. (B) Dot plot showing the distribution of filtered SNVs in gene coding region in ADHD children. Allele frequency and influence on protein functions were marked by dot size and color, respectively. (C) Dot plot showing the distribution of genes corresponding to SNVs in promoter region in ADHD children. Gene expression level and involved transcriptional regulators were marked by dot size and color, respectively. (D) Dot plot showing the distribution of genes corresponding to SNVs in 3'-UTR in ADHD children. Gene expression level and involved miRNAs were marked by dot size and color, respectively.

Fig.S2 Additional information of the distribution and annotation of filtered SNVs in dopamine related genes. (A) A heatmap showing the distribution of intersect genes in ADHD susceptible regions and dopamine related functions. Gene expression levels were indicated by colors. (B) Dot plot showing the distribution of genes corresponding to SNVs in 3'-UTR in ADHD children. Gene expression levels were marked by dot sizes. (C) Heatmaps showing the distribution of filtered SNVs in promoter regions in the ADHD children. Genotype categories of each site were labeled in different colors as indicated in the legend. (D) Dot plot showing the distribution of genes corresponding to SNVs in promoter region in ADHD children. Gene expression levels were marked by dot sizes.
