# Supplementary figures and images for: A Systematic Screening of ADHD-Susceptible Variants From 25 Chinese Parents–Offspring Trios
Source: Front Genet. 2022 Apr 26;13:878036. doi: 10.3389/fgene.2022.878036 (PMC9087589; doi:10.3389/fgene.2022.878036)

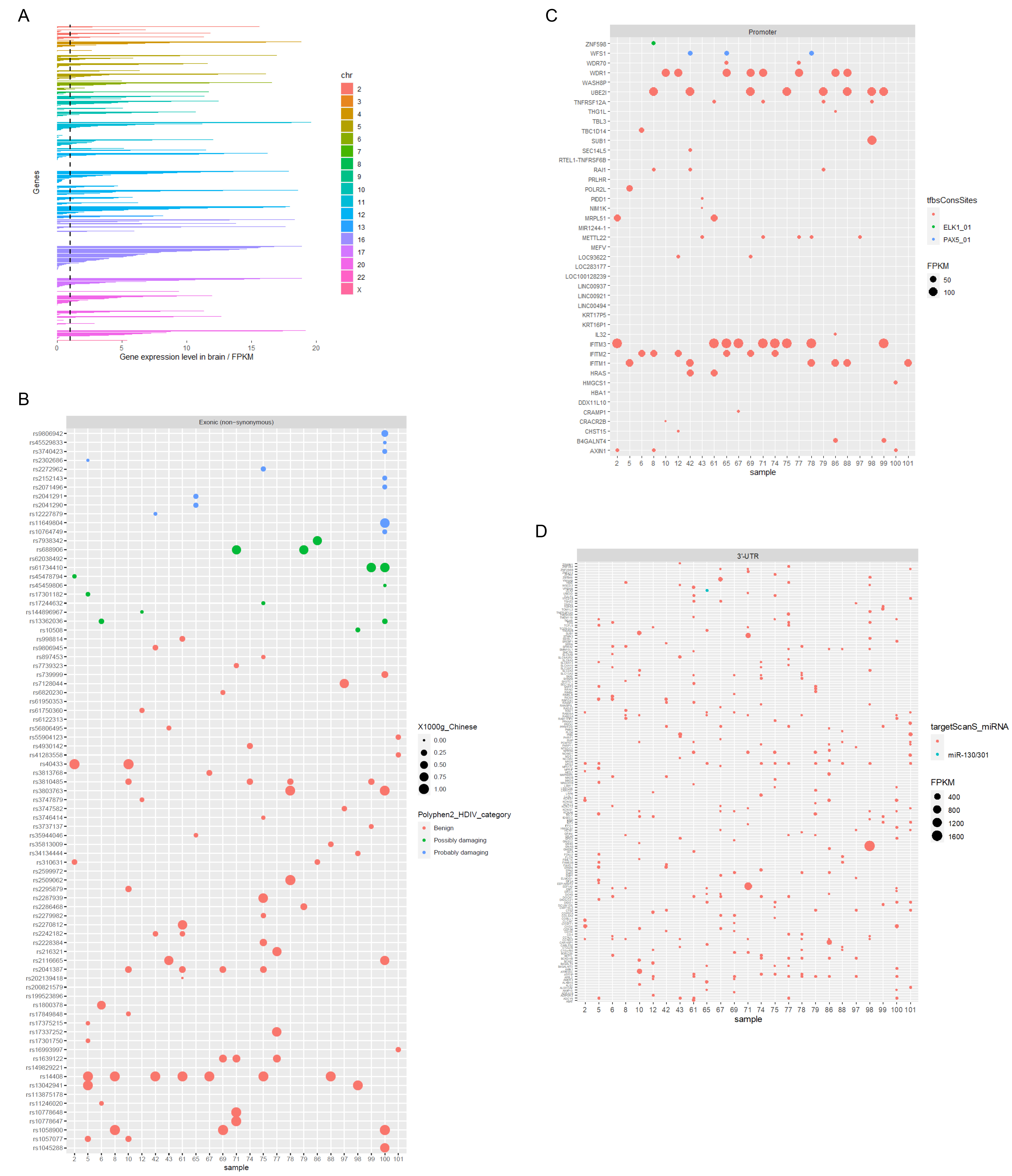

Supplement: Supplementary file 2 [file Image1.JPEG]

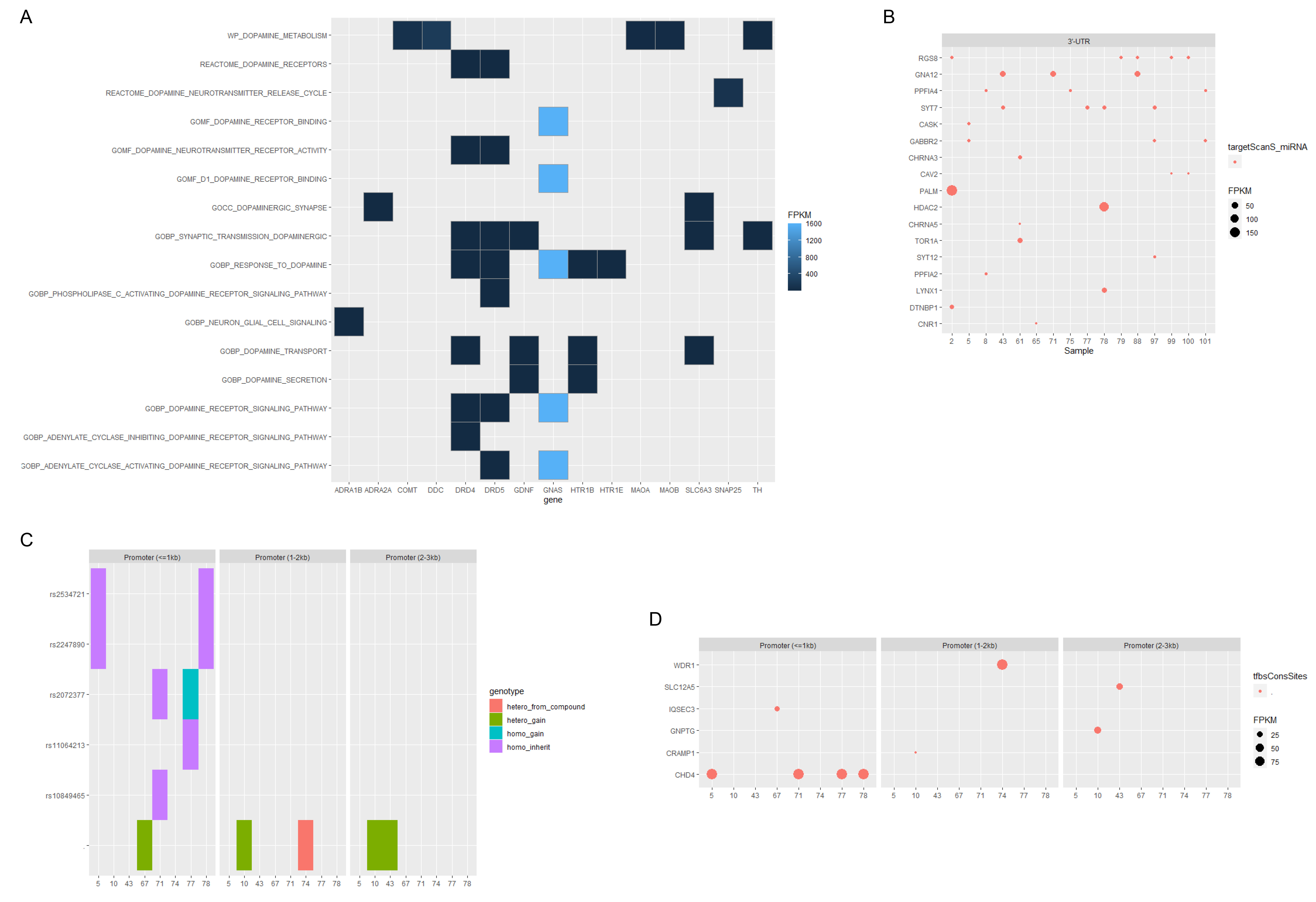

Supplement: Supplementary file 3 [file Image2.JPEG]
